# Syrian Hamster as an Animal Model for the Study on Infectious Diseases

**DOI:** 10.3389/fimmu.2019.02329

**Published:** 2019-10-01

**Authors:** Jinxin Miao, Louisa S. Chard, Zhimin Wang, Yaohe Wang

**Affiliations:** ^1^Department of Science and Technology, Henan University of Chinese Medicine, Zhengzhou, China; ^2^Sino-British Research Center for Molecular Oncology, National Center for the International Research in Cell and Gene Therapy, School of Basic Sciences, Academy of Medical Sciences, Zhengzhou University, Zhengzhou, China; ^3^Centre for Molecular Oncology, Barts Cancer Institute, Queen Mary University of London, London, United Kingdom

**Keywords:** infectious diseases, Syrian hamster, drug discovery, infection mechanism, biomedical research

## Abstract

Infectious diseases still remain one of the biggest challenges for human health. In order to gain a better understanding of the pathogenesis of infectious diseases and develop effective diagnostic tools, therapeutic agents, and preventive vaccines, a suitable animal model which can represent the characteristics of infectious is required. The Syrian hamster immune responses to infectious pathogens are similar to humans and as such, this model is advantageous for studying pathogenesis of infection including post-bacterial, viral and parasitic pathogens, along with assessing the efficacy and interactions of medications and vaccines for those pathogens. This review summarizes the current status of Syrian hamster models and their use for understanding the underlying mechanisms of pathogen infection, in addition to their use as a drug discovery platform and provides a strong rationale for the selection of Syrian hamster as animal models in biomedical research. The challenges of using Syrian hamster as an alternative animal model for the research of infectious diseases are also addressed.

## Introduction

According to data released by the World Health Organization (WHO), infectious agents causing lower respiratory infections, diarrheal diseases, and tuberculosis were ranked in the top ten causes of death worldwide, resulting in 5.7 million deaths in 2016 (1). It is clear that we need to improve our understanding of these diseases and pathogenic agents in order to develop more effective drugs and vaccines. To this end, we need a suitable animal model that can most accurately mimic the pathogenesis of infection as infection usually induces a complex process of host immune responses that *in vitro* experiments are unable to simulate. Only *in vivo* models can accurately assess the complexity of host responses and allow the efficacy and adverse effects of drugs or vaccine to be evaluated.

The Syrian hamster (*Mesocricetus auratus*) has been used as an animal model to study human-associated diseases for over 60 years. A number of studies have documented that Syrian hamsters represent better models for analysis of viral infections compared to murine models as the similarity to humans with regard to disease symptoms, pathognesis and immune responses is greater (2–4). It has been demonstrated by us and others that human cytokines, including granulocyte-macrophage colony-stimulating factor (GM-CSF) and interleukin-12 (IL-12), are fully functional in hamster models, but not in mouse models (5, 6). Together with other advantages, such as fast reproductive rate and ease of handling, Syrian hamsters are a superior choice compared with other small animals.

**Figure 1:**
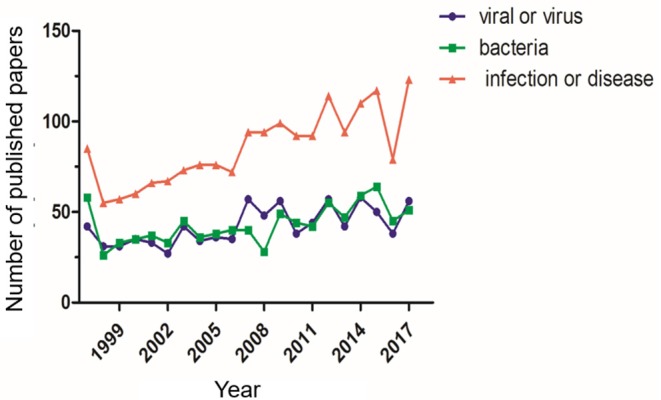
Number of publications using Syrian hamsters as a disease model. The number of publications using Syrian hamsters as an animal model from 1997 through 2017 is shown. For each standard, the number of publications was determined via a search using the ScienceDirect database. The search was performed with the keywords “Syrian hamster” or “golden hamster” AND “model” AND (1) “viral” or “virus,” (2) “bacteria,” (3) “infection” or “disease”.

Although Syrian hamsters have historically been used in diseases research, their value as an animal model in the study of infectious diseases has only recently been realized. With advancements in gene editing technologies, their popularity has increased significantly (Figure 1). The use of genetically engineered Syrian hamster (GESH) models is critical for understanding disease progression and for developing prophylactic and therapeutic treatment regimens. The first STAT2 gene knockout (KO) Syrian hamster was developed in 2014, using the CRISPR/Cas9 system to target the hamster germline (7). STAT2 is a crucial element of the type I interferon (IFN) signal transduction pathway and the hamster model has emerged as the only small animal model permissive for Adenovirus (AdV) infection, thus, the STAT2 KO model has been critical for the characterization of Adenovirus pathogenesis (8).

## Syrian Hamster Used for Research in Viral Infections

The Syrian hamster is an ideal small animal model to study the disease caused by virus infection. Previous studies have shown that some human-specific viruses can also infect, replicate and cause similar pathological alterations in Syrian hamsters (9). In particular, Syrian hamsters are recognized as valuable model for studying emerging and acute human viral diseases caused by highly pathogenic RNA viruses (10). Thus, these animals are of great value for testing potential vaccines and new therapeutic drugs for human use. At present, over 70 different viruses have been investigated using Syrian hamster, and more viral infection studies will be explored in the future (Table 1). In this review, we focus on six viruses for which the use of the Syrian hamster has provided valuable insight into disease pathogenesis.

**Table 1:** Viral infections in Syrian hamster models.

**Agent**	**Syrian hamster strain**	**Disease model**	**References**
**Paramyxoviruses**			
Nipah virus	WT	Nipah disease	(11)
Hendra virus	WT	Hendra disease	(12)
**Flaviviruses**			
West Nile virus	WT	West Nile neurological syndrome	(13)
Yellow fever virus^*^	WT	Yellow fever	(14)
Zika virus	STAT2^−/−^	Zika virus disease	(15)
St. Louis encephalitis virus	WT	Chronic St. Louis encephalitis^**^	(16)
Japanese encephalitis virus	WT	Japanese encephalitis	(17)
Eastern equine encephalitis virus	WT	Eastern equine encephalitis	(18)
Venezuelan equine encephalitis virus	WT	Venezuelan equine encephalitis^**^	(19)
Western equine encephalitis virus	WT	Western equine encephalitis	(20)
**Filoviruses**			
Ebola virus^*^	WT	Ebola virus disease	(21)
Marburg virus^*^	WT	Marburg virus disease	(22)
Marburg virus	STAT2^−/−^	Marburg virus disease	(23)
Crimean–Congo hemorrhagic fever virus	WT	Crimean Congo hemorrhagic fever	(24)
**Arenaviruses**			
Pichinde virus	WT	Lassa fever-like	(25)
Pirital virus	WT	Arenavirus disease	(26)
**Phleboviruses**			
Rift Valley fever virus	WT	Rift Valley fever	(27)
Heartland virus	STAT2^−/−^	HRTV disease	(28)
Punta Toro virus	WT	Rift Valley fever-like^*^	(29)
Gabek forest virus	WT	Rift Valley fever-like^*^	(30)
Severe fever with thrombocytopenia syndrome virus	STAT2^−/−^	Severe fever with thrombocytopenia syndrome	(31)
**Others**			
Andes virus	WT	Hantavirus pulmonary syndrome	(32)
Maporal virus	WT	Hantavirus pulmonary syndrome	(33)
SARS coronavirus	WT	severe acute respiratory syndrome^**^	(34)
Oncolytic adenoviruses	WT	Pancreatic cancer	(35)
Adenoviruses	RAG1^−/−^	Immunodeficiency disease	(36)
Adenoviruses	STAT2^−/−^	Immunodeficiency disease	(8)
Prions	WT	Scrapie disease	(37)

* *Adapted viruses used in model*.

** *Infection model, not disease model. WT, Wild-type*.

### West Nile Virus

The most intensively studied virus in Syrian hamsters is West Nile virus (WNV). WNV is a member of the genus *Flavivirus* (family *Flaviviridae*), an emerging zoonotic arbovirus widely distributed throughout the world (38). WNV is usually transmitted via bites on infected arthropods (mosquitos). In humans, the majority of WNV infections are asymptomatic, with only 20% of infected individuals developing symptomatic West Nile fever (WNF) (39). However, WNV is an important emerging neurotropic virus causing severe encephalitis in human posing a significant threat to global health (40). Syrian hamsters can be readily infected by mosquito bite, ingestion (oral) or needle inoculation and infected hamsters develop viremia and illness, with symptoms similar to those experienced during human infection (41, 42). Using this hamster model, Xiao et al. observed both histologic abnormalities and appearance of viral antigen in the brain first followed by the spinal cord, with infection eventually leading to acute central nervous system (CNS) injury (13). Infected hamsters developed neurological disease (43–46) and association of suppressed diaphragmatic electromyographs (EMGs) with infection of the medulla oblongata (47). Samuel et al. also found that inoculation of Syrian hamster with WNV resulted in paralysis of the hind limb ipsilateral but not contralateral to the injection site (48). Mateo et al. generated a model of immunosuppressed Syrian hamsters using cyclophosphamide and after infection the hamsters displayed similar clinical signs to those observed in an immunosuppressed cancer patient infected with WNV (49). By observing the pathogenesis of disease in WNV-infected immunocompromised hamsters, the animals were shown to develop chronic viremia and sustained renal infection for 8 months (50). Syrian hamsters not only display an adaptive immune response but also mount an innate immune response to WNV infection. Since the Syrian hamster has been shown to be a suitable model for WNV infection, it has also been used to test the efficacy of anti-WNV-neutralizing humanized monoclonal antibody, hE16 (44). Antibody immunoprophylaxis induced by delivery of recombinant antigens (WN-80E or WN-NS1) also protected Syrian hamster from WNV infection (51). Using a Syrian hamster model, Widman et al. successfully demonstrated that RepliVAX WN, a single cycle flavivirus vaccine platform, was able to induce durable protective immunity against WNV challenge (52). These studies demonstrate Syrian hamster as an ideal model for study of the pathogenesis of WNV infection and assessing new approaches for WNV treatment and prevention.

### Yellow Fever Virus

YFV is an arthropod-borne virus of the genus *Flavivirus* (family *Flaviviridae*) and has high morbidity and mortality rates in regions of sub-Saharan Africa and South America (53). It was one of the first viruses of humans to be identified, isolated, propagated *in vitro* and studied by genomic sequencing (54). The study of infection mechanism of YFV has historically been hindered by the lack of appropriate small animal model and non-human primate (NHP) models have typically been used. More recently, several research groups have generated animal models using Syrian hamsters that can be successfully infected with YFV (55–58). McArthur et al. reported adapted viral strains (Asibi/hamster p7) allow the reproduction of yellow fever disease in hamsters with features similar to the human disease (59). Further, studies have also shown that infection of Syrian hamster results in immune responses that correspond to those observed in infected humans, with marked increases in IFN-γ, IL-2, TNF-α in the spleen, kidney, and heart, but reduced levels of these seen in the liver. In addition, these studies found increased levels of IL-10 and reduced levels of TGF-β in the liver, spleen, and heart in early and mid-stages of infection (60). Syrian hamster can be used both to study the pathogenesis of the YFV infection, and to validate antiviral drugs and antiviral therapies. Recent findings have shown that treatment with the anti-viral compounds 2′-C-methyl cytidine (61), T-1106 (62), IFN alfacon-1 (63), and BCX4430 (64) pre- and post-YFV exposure can significantly improve Syrian hamster survival. In a study by Julander et al. immunization with DEF201, an AdV type-5 vector expressing IFN alpha (IFN-α), can effectively reduce the viral titer in hamster's liver and serum post-YFV infection (65). Immunoprophylaxis with XRX-001, a vaccine containing inactivated yellow fever antigen with an alum adjuvant, can elicit high titers of neutralizing antibodies *in vivo* to protect Syrian hamsters from YFV infection (66, 67). Interestingly, Xiao et al. (67) and Tesh et al. (68) demonstrate that prior exposure of Syrian hamsters to heterologous flaviviruses reduces the risk of YFV infection.

### Nipah Virus

Nipah is paramyxovirus of the genus *Henipavirus* (family *Paramyxoviridae*) with a high fatality rate (69). Infection in humans usually causes severe encephalitic and respiratory disease (70). After inoculation with Nipah virus (NiV), Syrian hamsters also develop characterisitic neurological disease (12). Similar to symptoms after human infection, pathological lesions are the most severe and extensive in the hamster brain and viral antigen and RNA can be detected in neurons (11), lung (71), kidney, and spleen (11). The Syrian hamsters in the majority of NiV infection studies are treated by intraperitoneal (IP) injection or intranasal (IN.) delivery and these models have revealed that different inoculation method can cause diverse pathological responses (11). In Wong's work, IP injection of NiV in Syrian hamsters caused primarily neurological disease, while IN delivery developed neurological symptoms as well as labored breathing due to lung infection in the final stages of disease (11). Disease progression is usually much rapid and the time to death post-infection is shorter following intraperitoneal rather than intranasal inoculation (72). Since the Syrian hamster has shown suitability for studying NiV infection, it was further used to study the viral transmission (73–75), demonstrating that Nipah virus is transmitted efficiently via direct contact and inefficiently via fomites, but not via aerosols. Regarding the use of these models for development of disease treatment and prophylaxis, recent studies have shown that pretreatment with Poly(I)-poly(C_12_U) can significantly decrease the mortality caused by NiV infection of Syrian hamster (76). In addition, the model was used as a platform for evaluation of vaccines for NiV (77–80). Walpita et al. discovered purified NiV-like particles (VLP) can protect the Syrian hamster using either multiple-dose or single-dose vaccination regimens followed by NiV challenge (81).

### Ebola Virus

Ebola virus (EBOV) is one of five known viruses within the genus *Ebolavirus* (family *Filoviridae*) (10). It's classified as biosafety level 4 (BSL-4) pathogen by the WHO. Not only can Syrian hamsters be effectively infected with mouse adapted (MA)-EBOV, they additionally display major hallmarks infection and pathogenesis seen in humans and non-human primates (NHPs). Syrian hamsters could be inoculated *via* intraperitoneal injection with mouse adapted Zaire Ebola virus (MA-ZEBOV). The pathology caused by this infection is similar to that of humans, which includes significant spleen and liver damage, cytokine dysregulation, severe coagulopathy, lymphocyte apoptosis, and infected organ necrosis or apoptosis (21, 82). The immune responses of infected Syrian hamsters include activation of T cell and antibody production. In a recent study, the results of Ebola virus infection in hamsters demonstrate that CD4^+^ T cells are required for natural immunity and CD4-dependent antibody responses are required for immunity against the virus in this model (83). Syrian hamsters can be used to evaluate a bivalent vaccine comprising recombinant Vesicular stomatitis virus (VSV) expressing two different immunogens derived from ZEBOV envelope glycoprotein (84) and Andes Virus (ANDV) (32). The results showed that a single immunization with this vaccine provides hamsters complete and sterile protection against lethal challenge with MA-ZEBOV or ANDV (85).

### Marburg Virus

Marburg virus (MARV) is also a negative sense RNA virus belonging to the family *Filoviridae* that causes hemorrhagic fever (86). Researchers have shown that Syrian hamsters can be used to study MARV infection. The Syrian hamster model was established to study MARV infection using the Angola variant (HA-MARV) (22). In the study, hamsters inoculated with HA-MARV developed hemorrhagic manifestations, coagulation abnormalities, dysregulation of pro-inflammatory chemokines MIP-1α and IP-10, and increment of type I interferon responses (22, 87). In addition, Atkins et al. recently developed a small animal model for wild-type MARV infection using STAT2 KO Syrian hamster, in which viral replication rapidly progresses to multiorgan infection and extensive viremia (23), demonstrating STAT2 as a key host factor affecting wild-type MARV infection.

### Rift Valley Fever Virus

RVFV is a member of the *Bunyaviridae* family and the genus *Phlebovirus* (88, 89). RVFV is usually transmitted via bites of infected mosquitos and can lead to mild febrile illness, retinitis, fulminant hepatitis, encephalitis and viral hemorrhagic fever (90). The infection of RVFV in Syrian hamsters has been well-described (91). The study results have assessed the susceptibility of Syrian hamsters to RVFV infection and shown that viral infection results in viremia, elevation of viral loads in liver, brain, and spleen tissues, observation of severe hepatocellular necrosis in the early stage of infection, and intense immunoreactivity of affected hepatocytes (27, 92, 93). Furthermore, using Syrian hamsters, Scharton et al. proved that prophylactic Favipiravir (T-705) can effectively protect infected individuals against RVFV infection and reduce delayed-onset neurologic disease observed with ribavirin treatment (94). In another study, Gowen et al. used hamsters to demonstrate protection from infection with just a single-dose intranasal treatment of the AdV-IFNα vector DEF201 (95). In addtion, results presented by Westover et al. demonstrate that the adenosine analog, galidesivir (BCX4430), can effectively reduce the RVFV titer in infected Syrian hamsters (96).

### Other Viruses

A large number of other studies have also demonstrated that Syrian hamster is a permissive small animal model for other viruses, for example, Syrian hamster model was successfully used to test the efficacy of anti-F MAbs to reduce Hendra virus infection (12). STAT2 KO Syrian hamster have shown successful infection with Zika virus (ZIKV) and the infected hamsters displayed the similar symptoms as in human (15). Also, an immunosuppressed Syrian hamster generated by Schaecher et al. strengthened its valuable application in study of severe acute respiratory syndrome coronavirus (SARS-CoV) infection (34). Syrian hamster has successfully been characterized for infection of human influenza, including the recent H1N1, pdm09, and H3N2 viruses (97). Moreover, as a permissive immunocompetent animal model for the study of oncolytic adenovirus, its use has been expanding for the study of cancer virotherapies (98–100).

## Syrian Hamster Used for Research in Bacterial and Parasitic Infections

Syrian hamster is also an ideal animal model for the study of a series of human bacterial and parasite infections, and its application has been well-reported in the literature (Table 2). Among the pathogens studied, some, such as Babesiosis, Leptospirosis, and Leishmaniasis can cause fatal infection. As for studies on virus pathogenesis, the value of the Syrian hamster model is not only reflected in the study of pathological and immune response to these infections, but also in the discovery of potential drugs and treatments.

**Table 2:** Major bacterial and parasitic infection studies in the Syrian hamster.

**Agent**	**Syrian hamster strain**	**Disease model**	**References**
*Clostridium difficile*	WT	*Clostridium difficile* disease	(101)
Leptospira	WT	Leptospirosis	(102)
Helicobacter spp.	WT	Helicobacter spp. disease	(103)
*Entamoeba histolytica*	WT	Amebic liver abscess	(104, 105)
Leishmania	WT	Visceral Leishmania	(106)
Babesia	WT	Babesiosis	(107)

### Leptospira interrogan

Pathogenic *Leptospira interrogans* is spirochete bacteria responsible for leptospirosis, a widespread and emerging neglected zoonotic. Syrian hamster is the preferred model to study the infection of serovars of *Leptospira interrogans*, with bacteria traveling rapidly to the bloodstream via the lymphatics, then invading tissues and infecting all organs prior to the death of the hamsters (108). Similar to human, the presence of *Leptospira interrogans* can be detected in liver and kidney, with the destruction of hepatocyte junctions that leads to jaundice, thrombotic glomerulopathy, and interstitial nephritis (109, 110). Infected animals develop the enhanced expression of pro-inflammatory cytokines by peripheral blood cells, such as IL-1α, IL-10, TNF-α (111). Dramatic imbalance in the cytokine production upon Leptospira infection might play an important role in the development of severe leptospirosis (112). Since the Syrian hamster has been shown to be a suitable model, it has been used to test the efficacy of vaccines against this disease. Palaniappan et al. demonstrate that the immunization of Syrian hamster with recombinant LigA (rLigA) prevents fatalities, with decreased histopathological lesions in kidney and inhibited the growth of the organisms (113). In another study, a vaccine using a conserved region of the leptospiral immunoglobulin-like B protein (LigB, 131–645) and aluminum hydroxide (AH) can significantly increase IgG and IgM levels in the hamster, protecting the animal from mortality after challenge (114).

### Clostridium difficile

*Clostridium difficile* disease caused by *Clostridium difficile* infection (CDI) is one of the most common infectious diseases worldwide (115). The increasing threat of morbidity and mortality caused by the infection is mostly due to the emergence of hypervirulent strains, increased use and misuse of antibiotics (116). The use of mouse animal model has been unable to provide CDI drug discoveries, so it is necessary to find new animal models (117, 118). Several groups have generated Syrian hamster models for CDI, which developed many of clinical symptoms observed in infected humans (119–121). In these studies, Syrian hamsters were conditioned with a single subcutaneous injection of clindamycin to induce *Clostridium difficile* colitis model. Using this infection model, the efficacy of LFF571 antibiotic against *Clostridium difficile* was assessed (122) and the oral mixture of kefir-isolated bacteria and yeasts to prevent diarrhea and enterocolitis triggered by *Clostridium difficile* was tested (123).

### Leishmania donovani

Visceral leishmaniasis (VL; also known as kala-azar) is the most severe form of leishmaniasis caused by *Leishmania donovani* and *Leishmania infantum* (*Leishmania chagasi* in the Americas) (124). The Syrian hamster is highly susceptible to infection with visceralizing *Leishmania* species and is considered the best experimental model to study VL as it reproduces the clinicopathological features of human disease and quite distinct from those noted in murine models of infection (125). In the majority of studies, animals were infected by the intracardial route. Infected animals demonstrate up-regulated expression of Th1-associated cytokine mRNA, such as IFN-γ, IL-2, and TNF-α in the spleen, but limited induction of IL-4 mRNA (126). In murine models, *Leishmania* is controlled through nitric oxide (NO) generation, and however in hamsters, as in humans, NO does not have a role in macrophage function. Inducible NO synthetase (iNOS) mRNA was not detected in livers or spleen of hamsters, which may explain the uncontrolled parasite replication occurring in hamsters and humans, despite the induction of a strong Th1 cytokine response (126). Not only can Syrian hamster model be used to study the pathogenesis of *Leishmania donovani* infection, but also to test vaccines as recent studies have shown. Kushawaha et al. used a Syrian hamster model to show that recombinant *Leishmania donovani* protein disulfide isomerase (rLdPDI) generated a robust cellular immune response with increased iNOS transcription and TNF-α, IFN-γ, and IL-12 levels (127). In another study by Samant et al. vaccination with DNA-encoding N-terminal domain of the PPG gene in golden hamsters yielded 80% protection against *Leishmania donovani* challenge with generation of Th1 type of immune response (128).

### Leishmania infantum

Besides *Leishmania donovani, Leishmania infantum* has also been studied using the Syrian hamster model. Moreira et al. generated a model using Syrian hamsters featuring a similar human clinical picture on *Leishmania infantum* infection (129). The animals developed hepatosplenomegaly, severe weight loss, anemia, and leucopenia. A study found increased levels of IgG in hamsters infected with *Leishmania infantum* (130). Similar to humans, Syrian hamsters can develop the progressive fatal disease, with major sites of parasites replication being the liver, spleen, and bone marrow, eventually causing death of the host (131). Infection of the hamsters showed a strong humoral response against Leishmania antigens, and high antibody levels (131). Study have tested the LJM19 (Immunization with 16 DNA plasmids coding for salivary proteins of *Lu. longipalpis*) protein protected hamsters against the fatal outcome of VL (132).

### Entamoeba histolytica

*Entamoeba histolytica* is a popular protozoan parasite causing amebiasis in humans that is a major source of morbidity and mortality in the developing countries (133). Parasitic *Entamoeba histolytica* produces amebic colitis and an amebic liver abscess (ALA). Syrian hamster can be successfully infected with *Entamoeba histolytica* (104). Similar to symptoms after human infection, the main extraintestinal complication, ALA, is also found in the hamster. In the hamster, liver recruitment of neutrophils is the initial host response to *Entamoeba histolytica* infection (134). A study indicated that leukocytes can induce *Entamoeba histolytica* trophozoites to undergo cell death (135). Although an anti-parasitic drug (Metronidazole) exists, side effects of toxicity exist in patients; thus this model has been used to develop alternative therapeutic agents. One research group showed that bovine lactoferrin protected against hepatic amoebiasis in Syrian hamster model (136). Hamsters were also used to show that intraperitoneal injection of *Entamoeba histolytica* surface metalloprotease (EhMSP-1), an antigen vaccine, protected against the amebic liver abscesses (137). In this study, EhMSP-1 immunization stimulated a robust IgG antibody response, IgG bound to the surface of *Entamoeba histolytica* trophozoites and accelerated amebic lysis via activation of the classical complement cascade. The same animal model used for *Entamoeba histolytica* infection was used to show that baculovirus driving the expression of the Gal-Lectin LC3 fragment, when administeredvia intramuscular injection, increased IFN γ and IL-4 levels in the liver to protect against ALA (138).

### Schistosoma haematobium

*Schistosoma haematobium* (urinary blood fluke) is the etiologic agent for urogenital schistosomiasis, a source of morbidity and mortality for over 112 million people worldwide (139). Although an improved mouse model of *S. haematobium* urinary tract infection can recapitulate several aspects of human urogenital schistosomiasis (139), Syrian hamsters still show advantages compared to mouse. Syrian hamsters can be transdermally infected with *Schistosoma haematobium* cercariae (140, 141). A model with *Schistosoma haematobium* cercariae granulomatous-like immune reaction and hepatic fibrosis infection using Syrian hamsters was generated by Botelho et al. (142). Botros et al. used Syrian hamster model to analyze and test praziquantel (PZQ) treatment (143). This animal model revealed predominant CD4^+^ T cells in the acute phase of granuloma formation in the liver [75 days post-infection (PI)], Confluent granulomata with multiple eggs in the center were observed in the liver and urinary bladder with the preponderance of CD8^+^ positive T cells in the liver (95 and 115 days PI). In this model, high dose PZQ was clearly curative from 75 days PI.

### Others

There are many studies detailing the pathogenesis of other bacteria and parasitic infections using the Syrian hamster animal model that cannot be discussed here in detail. A Syrian hamster model to study *Borrelia burgdorferi* infection was established by Johnson et al. (144). After *Borrelia burgodrferi* infection, hamsters were utilized to study articular manifestations of Lyme borreliosis, which is similar to human (145). Syrian hamster can be successfully infected with *Leishmania* panamensis (146). Infected animals have up-regulated expression of type II cytokines (IL-4 and IL-13), down-regulation of IL-12, and up-regulation of the type II chemokine CCL17 and its receptor CCR4 in lymph node. Grogl et al. generated a model using Syrian hamsters for drug discovery for *Leishmania panamesis* infection (147). After *Leishmania braziliensis* infection, Ribeiro-Romao et al. observed large and ulcerated lesions with elevated levels of interferon-γ and tumor necrosis factor (TNF) during the infection endpoint, which suggests that these cytokines contribute to tissue injury (148). Treatment of *Leishmania amazonensis* infection by intralesional administration of dimethyl carbaporphyrin ketal (CKOMe) reduced the parasite load without noticeable toxic effects in liver (149). A Syrian hamster model to study *Plasmodium berghei* infection (150) demonstrated induction of severe malaria in the Syrian hamster window chamber model and was used to investigate microcirculatory changes and tissue oxygenation (151). The reader is referred to the relevant publications for further information regarding the use of Syrian hamster models to investigate these infections.

## Concluding Remarks

In this review, we described the use of the Syrian hamster model as an extraordinarily effective and relevant platform for evaluation of the molecular mechanisms of immune responses to infectious diseases. These studies focus on several infectious pathogens including those of viral, parasitic, and bacterial origins. The results indicate that the Syrian hamster immune response is more physiological similar to the human immune response when compared to other animals, thus offering unique advantages when studying the disease pathogenesis and for novel drug and treatment discovery. Future studies should consider determining additional similarities between the Syrian hamster and human immune response activation through pathogen manipulation of host metabolism. Increased research efforts will ultimately allow for the development of new technologies and tools to study the Syrian hamster, such as more accurate sequencing technology along with specific antibodies against hamster proteins that are currently limited in comparison to similar tools for studying murine responses to infection. We believe that the recent advances that the Syrian hamster model has contributed enormously to our understanding of infectious diseases and disease management and demonstrates the strong potential for future research and development of anti-viral drug discovery.

However, as discussed, the lack of research tools represents a major barrier to effective use of Syrian hamster models. Immunologic reagents for examing host immune response and particular gene expression, and transgenic disease models will all be required for a more complete evaluation of the value of this model. To overcome this, research groups are developing or identifying a considerable number of antibodies against Syrian hamster (Table 3) and hamster specific quantitative real-time PCR (RT-qPCR), transcriptome analysis and microarrays have also been developed (169). Most strikingly, CRISPR/Cas9 technology has rapidly sped up the creation of transgenic Syrian hamster disease models (170). These tools will overcome the limitations to research using Syrian hamsters, opening up a powerful platform for recapitulation of human disease pathogensis.

**Table 3:** List of antibodies tested in Syrian hamster.

**Gene**	**Antibody**	**Applications**	**References**
Apaf-1	Anti-Apaf-1 antibody	WB	(152)
Bax	Anti-Bax antibody	WB, IHC	(152)
Bcl-2	Anti-Bcl-2 antibody	WB, IHC	(152)
Bcl-xL	Anti-Bcl-xL antibody	WB	(153)
Caspase-2L	Anti-Caspase-2L antibody	WB	(152)
Caspase-3	Anti-Caspase-3 antibody	WB, IHC	(152)
Caspase-6	Anti-Caspase-6 antibody	WB	(152)
Caspase-8	Anti-Caspase-8 antibody	WB	(152)
Caspase-9	Anti-Caspase-9 antibody	WB	(152)
Cathepsin D	Anti-Cathepsin D antibody	WB, IHC	(154)
CD3	Anti-mouse or Syrian hamster CD3 (4F11) antibody	IHC, Flow Cyt	(155, 156)
CD4	Anti-mouse CD4 (GK1.5) antibody	IHC, Flow Cyt	(157, 158)
CD8β	Anti-rat CD8b (341) antibody	Flow Cyt	(158, 159)
CD20	Anti-CD20 antibody	IHC	(23)
CD25	Anti-CD25 antibody	Flow Cyt	(160)
CD68	Anti-CD68 antibody	IHC	(161)
COX-2	Anti-COX-2 antibody	WB, IHC	(162)
Cytochrome C	Anti-Cytochrome C antibody	WB	(152)
Fas	Anti-Fas antibody	WB	(152)
IL-4	Anti-IL-4 antibody	Flow Cyt	(160)
IFN-γ	Anti-IFN-γ-antibody	Flow Cyt	(160)
iNOS	Anti-iNOS antibody	IHC	(153)
ICAM-1	Anti-ICAM-1 antibody	WB	(163)
IκB	Anti-IκB antibody	WB	(152)
iNOS	Anti-iNOS antibody	WB	(153)
JAK2	Anti-JAK2 antibody	WB	(153)
lba-1	Anti-Iba-1 antibody	IHC	(159)
MARCO	Anti-hamster MARCO (PAL-1) antibody	IHC, Flow Cyt	(164)
Mcl-1	Anti-Mcl-1 antibody	WB	(165)
MHC II	Anti-mouse I-E^k^ MHC II (14-4-4S) antibody	Flow Cyt	(166)
MMP	Anti-MMP antibody	WB	(154)
MMP-2	Anti-MMP-2 antibody	WB, IHC	(152, 167)
MMP-9	Anti-MMP-9 antibody	WB	(152)
NF Kb-p50	Anti-NF Kb-p50 antibody	WB	(152)
NF Kb-p65	Anti-NF Kb-p65 antibody	WB	(152)
p-Akt	Anti-p-Akt antibody	WB	(154)
p-Ert	Anti-p-Ert antibody	WB	(154)
p-p65	Anti-p-p65 antibody	WB	(153)
p-STAT3	Anti-p-STAT3 antibody	WB, IHC	(154, 165)
p21^waf−1^	Anti-p21^waf−1^ antibody	WB	(152)
p53	p53 antibody	WB, IHC	(152, 168)
p65	p65 antibody	WB, IHC	(153)
PARP	Anti-PARP antibody	WB	(165)
Procaspase 3	Anti-Procaspase 3 antibody	WB	(153)
Procaspase 8	Anti-Procaspase 8 antibody	WB	(153)
Procaspase 9	Anti-Procaspase 9 antibody	WB	(153)
RAG1	Anti-RAG-1(D-5) antibody	WB	(36)
STAT2	Anti-STAT2 antibody	WB	(7)
STAT3	Anti-STAT3 antibody	WB	(153)
Survivin (C)	Anti-Survivin (C) antibody	WB	(165)
Survivin (N)	Anti-Survivin (N) antibody	WB	(165)
TNF-α	Anti-mouse TNF α antibody	WB	(154)
TRAF1	Anti-TRAF1 antibody	WB	(153)
Ubiqutitin	Anti-Ubiqutitin antibody	WB	(154)
VCAM-1	Anti-VCAM-1 antibody	WB, IHC	(161, 163)

## Author Contributions

JM, ZW, and YW: manuscript concept and design. JM: manuscript writing. LC, ZW, and YW: manuscript revising.

### Conflict of Interest

The authors declare that the research was conducted in the absence of any commercial or financial relationships that could be construed as a potential conflict of interest.
